# Potential Mitigation of Smoke Taint in Wines by Post-Harvest Ozone Treatment of Grapes

**DOI:** 10.3390/molecules26061798

**Published:** 2021-03-23

**Authors:** Margherita Modesti, Colleen Szeto, Renata Ristic, WenWen Jiang, Julie Culbert, Keren Bindon, Cesare Catelli, Fabio Mencarelli, Pietro Tonutti, Kerry Wilkinson

**Affiliations:** 1 Life Sciences Institute, Scuola Superiore Sant’Anna, Piazza Martiri della Libertà 33, 5612 Pisa, Italy; pietro.tonutti@santannapisa.it; 2 Department of Wine Science, Waite Research Institute, The University of Adelaide, PMB 1, Glen Osmond, SA 5064, Australia; colleen.szeto@adelaide.edu.au (C.S.); renata.ristic@adelaide.edu.au (R.R.); kerry.wilkinson@adelaide.edu.au (K.W.); 3 The Australian Research Council Training Centre for Innovative Wine Production, PMB 1, Glen Osmond, SA 5064, Australia; 4 The Australian Wine Research Institute, P.O. Box 197, Glen Osmond, SA 5064, Australia; maddy.jiang@awri.com.au (W.J.); julie.culbert@awri.com.au (J.C.); keren.bindon@awri.com.au (K.B.); 5 P.C. di Pompeo Catelli S.R.L., Via Roma 81, Uggiate Trevano, 22029 Como, Italy; cesare.catelli@pinco-sa.com; 6 Department of Agriculture Food and Environment, University of Pisa, Via del Borghetto 80, 56124 Pisa, Italy; fabio.mencarelli@unipi.it

**Keywords:** amelioration, glycoconjugates, rate-all-that-apply, smoke taint, volatile phenols, wine

## Abstract

When bushfires occur near grape growing regions, vineyards can be exposed to smoke, and depending on the timing and duration of grapevine smoke exposure, fruit can become tainted. Smoke-derived volatile compounds, including volatile phenols, can impart unpleasant smoky, ashy characters to wines made from smoke-affected grapes, leading to substantial revenue losses where wines are perceivably tainted. This study investigated the potential for post-harvest ozone treatment of smoke-affected grapes to mitigate the intensity of smoke taint in wine. Merlot grapevines were exposed to smoke at ~7 days post-veraison and at harvest grapes were treated with 1 or 3 ppm of gaseous ozone (for 24 or 12 h, respectively), prior to winemaking. The concentrations of smoke taint marker compounds (i.e., free and glycosylated volatile phenols) were measured in grapes and wines to determine to what extent ozonation could mitigate the effects of grapevine exposure to smoke. The 24 h 1 ppm ozone treatment not only gave significantly lower volatile phenol and volatile phenol glycoside concentrations but also diminished the sensory perception of smoke taint in wine. Post-harvest smoke and ozone treatment of grapes suggests that ozone works more effectively when smoke-derived volatile phenols are in their free (aglycone) form, rather than glycosylated forms. Nevertheless, the collective results demonstrate the efficacy of post-harvest ozone treatment as a strategy for mitigation of smoke taint in wine.

## 1. Introduction

In recent years, climate change has resulted in hotter and drier summers and as a consequence, the frequency and severity of bushfires is increasing [1,2]. Wine producing countries including Australia, Canada, Chile, Portugal, South Africa, New Zealand and the US, have endured significant economic losses due to smoke and/or fire damage from bushfires burning in or near grape growing regions [2,3,4,5]. In the case of vineyard exposure to bushfire smoke, wines made from smoke-affected grapes can exhibit smoky, medicinal and ashy characters, and are therefore defined as “smoke tainted” [6,7,8].

The chemical and sensory consequences of smoke taint have been attributed to several volatile phenols (VPs) which are derived from thermal degradation of lignin during the combustion of plant material [9]. VPs can be taken up by grapevine leaves and berries [4,8,10,11]; the most abundant VPs detected in smoke-affected grapes and wines are guaiacol, 4-methylguaiacol, *o*-cresol and syringol [11,12,13,14]. VPs are toxic for plant cells and are thought to be transformed into glycosidic forms to prevent cellular damage [15,16]. The glycosylation process has been shown to occur rapidly following smoke exposure [17,18] and while VP glycoconjugates are odorless and flavorless, they can be metabolized during fermentation [11,13] and by human salivary enzymes [19], resulting in the release of VPs and perception of the smoke-related aromas and flavors, and ashy aftertaste characteristic of smoke taint.

Smoke taint has become an issue of increasing concern for grapegrowers and winemakers around the world since it is difficult to produce high-quality wine from smoke-affected grapes [3]. Over the past decade, considerable research has been undertaken to understand the impacts of grapevine exposure to smoke, and to mitigate the sensory consequences of smoke taint in wine; from partial defoliation of grapevines [20] and washing smoke-affected fruit [3,14,18] in the vineyard, to limiting skin contact during fermentation [7], and treating smoke-tainted wine via reverse-osmosis and solid phase adsorption [21] or the addition of activated carbon [22] or cyclodextrins [23], in the winery. These methods are often time and resource intensive and/or offer limited efficacy (i.e., they typically remove VPs, but not VP glycosides). The need for strategies that better mitigate the effects of smoke on grapes and wine is therefore imperative.

Ozone (O_3_) is used in food and beverage production as an environmentally friendly sanitizing agent, based on its strong oxidant properties, and it is classified as “generally recognized as safe” by the US Food and Drug Administration. Ozone is often applied to fruit and vegetables to reduce pathogen development thereby increasing the shelf life of fresh products [24,25]. Furthermore, in contrast with other sanitizers, O_3_ is rapidly reconverted to oxygen, achieving sanitization without chemical residues remaining on the treated products or in the environment [26,27]. Several applications have been proposed for the use of O_3_ at different stages of winemaking [28,29,30]. Post-harvest O_3_ treatment of grapes (controlled hyper-oxygenation) has been used to make sulfur dioxide-free wine (Purovino^®^ method, PC Engineering, Uggiate Trevano, Italy [31]) and to protect berries during post-harvest-controlled dehydration [32]. Although its application is typically limited to disinfection of surfaces, some important effects have been observed, including on the aroma profiles of fruit and vegetables. Post-harvest exposure to high doses of O_3_ has led to decreased concentrations of volatile compounds in multiple fruit species and cultivars, and this has been attributed to the oxidative effect of O_3_ [33,34,35]. Other studies suggest O_3_ might trigger defensive biochemical mechanisms in fruit that modify secondary metabolite profiles [25,36,37]. The fruit flavors of Petit Verdot grapes were enhanced following overnight treatment with a low dose of O_3_ [28], whereas short and repeated treatments of low concentrations of O_3_ caused a loss of aroma in grapes used in the production of Amarone wine [38]. These studies suggest the timing and concentration of O_3_ applied to grapes directly influences their volatile aroma composition.

Depending on the method of application, O_3_ can play dual roles: as an elicitor, stimulating the biosynthesis of important bioactive compounds (e.g., polyphenols and volatile compounds) [39]; or as an oxidizing agent, resulting in degradation of grape secondary metabolites [40]. Considering the oxidizing properties of O_3_ and its influence on grape and wine aroma, this study sought to evaluate the potential for post-harvest O_3_ treatment of smoke-affected grapes to be used as a novel strategy for mitigating smoke taint in wine. The aims of the study were therefore: (i) to evaluate the consequences of post-harvest O_3_ treatment of smoke-affected grapes on wine composition and sensory profiles; (ii) to determine to what extent post-harvest O_3_ treatment impacts grape color and phenolic composition; and (iii) to establish the influence of the duration and dose of O_3_ treatment on the concentration of smoke taint marker compounds in grapes and wine.

## 2. Results and Discussion

Research aims were achieved via two trials (each performed in triplicate) involving: (i) exposure of Merlot grapevines to smoke (for 1 h, at approximately 7 days post-veraison), then post-harvest O_3_ treatment of grapes (at 1 ppm for 24 h or 3 ppm for 12 h); (ii) post-harvest exposure of Merlot grape bunches to smoke, followed by O_3_ treatment at different dose rates (1 vs. 3 ppm), sampled at different times (6, 12 and 24 h). In the first trial, the concentration of smoke taint marker compounds (i.e., VPs, in free and glycosylated forms) were determined in control and smoke-affected Merlot grapes, before and during ozonation. This trial was subsequently taken through to a winemaking outcome and the composition and sensory profiles of the resulting wines determined. This enabled investigation of the impact of O_3_ treatment on the perceived intensity of smoke taint in wine. The second trial then evaluated the compositional effects of O_3_ applied to grapes immediately after smoke exposure, i.e., before glycosylation of smoke-derived volatile phenols. The impact of O_3_ on the phenolic compounds responsible for wine color and mouthfeel was also investigated in each of the trials.

### 2.1. Influence of Post-Harvest O_3_ Treatment on the Composition of Grapes Exposed to Smoke at Approximately 7 Days Post-Veraison

The concentrations of VP glycoconjugates observed in control and smoke-affected grapes are shown in Table 1; VPs were not detected in any grape sample (data not shown). Low levels of VP glycosides (<25 µg/kg) were detected in control grapes, irrespective of any O_3_ treatment, in agreement with previous studies which report VP glycosides as natural constituents of some grape varieties [11,13,41]. Grapevine exposure to smoke resulted in significantly higher levels of VP glycosides, particularly glycosides of guaiacol, phenol, cresols and syringol (Table 1), for which pentose glucosides of guaiacol, phenol and cresol and the glucose glucoside (gentiobioside) of syringol were most abundant (Table S1), again in agreement with previous research [18,42,43]. Statistically significant compositional differences were observed amongst smoke-affected grape samples. Interestingly, the highest VP glycoside concentrations were not detected in the smoke-affected grapes without O_3_ treatment, but in smoke-affected grapes treated with 3 ppm of O_3_ for 6 h. After 12 h of O_3_ treatment at 3 ppm, glycoside concentrations had declined significantly (*p* < 0.001), but were only marginally lower (i.e., ~5–9%) than the levels observed in smoke-affected grapes without O_3_ treatment (Table 1), and differences were not statistically significant. In contrast, the VP glycoside levels of smoke-affected grapes treated with 1 ppm of O_3_ were not significantly different after 6 or 12 h, but moderate differences (i.e., 12–20% decreases) were observed after 24 h of O_3_ treatment (Table 1). These results were consistent with a recent study which demonstrated the potential for ozonation to decrease the VP glycoside levels present in smoke-affected grapes [44].

The contrasting results obtained for 1 vs. 3 ppm applications of O_3_ were not completely unexpected, given that the effects of O_3_ on grape physiology and wine quality are known to be influenced by both the concentration and duration of ozonation [29,45,46,47,48], and likely reflect two different, but related mechanisms: oxidative stress and oxidation.

Plant tissue responds rapidly to O_3_ and within ~2 h of exposure, ozonation induces the formation and accumulation of different reactive oxygen species (ROS), mainly hydrogen peroxide, in the cell wall and plasma membrane [49]. When ROS production exceeds the capacity of scavenging systems to maintain the optimal redox status, oxidative stress occurs [50,51]. The oxidative stress induced by O_3_ exposure can stimulate cellular defense mechanisms, including biosynthesis of antioxidants such as glutathione, ascorbate and polyphenols [52,53,54,55]. Another defense mechanism triggered by oxidative stress is the increased activity of various enzymes, including uridine5′-diphospho-gluconosyltransferases (UGTs), which play indirect roles in ROS-removal [56]. Glycosylation (and deglycosylation) of antioxidants and phytohormones plays an important role in plant defense mechanisms [57]. Oxidative stress induces UGT activity, which can in turn increase glycosylation processes [51], and this might explain the observed increase in VP glycosides following 3 ppm O_3_ treatment of smoke-affected grapes (at t = 6). Glycosylation of smoke-derived VPs likely reflects a detoxification strategy, i.e., glycosylation allows compartmentalization and stable storage of small and toxic/reactive molecules, such as VPs, by lessening their volatility through derivatization [15].

In contrast, following 1 ppm O_3_ treatment of smoke-affected grapes, the concentration of VP glycosides declined. Rather than oxidative stress, the lower O_3_ dose rate may have resulted in partial oxidation of VP glycosides. Previous studies have reported modification of grape and/or wine aroma profiles as a consequence of oxidation due to ozonation [35,39,58], albeit the results reported in the literature are often contradictory. The outcome of ozonation of grapes strongly depends on grape variety, phenology and environmental conditions, in addition to O_3_ concentration, exposure time and application [29,45,46,47,48]. Nevertheless, in the current study, grape compositional data provided evidence to support some degree of mitigation of smoke taint as a consequence of post-harvest O_3_ treatment (at 1 ppm for 24 h).

### 2.2. Influence of Post-Harvest O_3_ Treatment on the Composition and Sensory Profiles of Wines Made from Grapes Exposed to Smoke at Approximately 7 Days Post-Veraison

Both grapevine smoke exposure and post-harvest O_3_ treatment slightly affected the basic compositional parameters of wines (Table 2). Wine made from smoke-affected grapes tended to have lower pH, alcohol content and color hue compared to control wine, whereas O_3_ treatment of control and smoke-affected grapes resulted in a small but significant decrease in the total phenolics content of wines. This was somewhat unexpected as previous studies have reported an increase in total phenolics for wine made from ozonated grapes (Petit Verdot and Sauvignon Blanc) [28,59], attributed to increased accumulation of antioxidants (such as polyphenols) in response to the oxidative stress induced by O_3_ [30,32]. In the current study, no differences were observed amongst grape anthocyanin profiles, regardless of smoke exposure or O_3_ treatment (Table S2). Nevertheless, the differences in basic wine chemistry that were observed were small and were therefore not expected to perceivably impact wine sensory properties.

Low levels of guaiacol and syringol (1 and ~3 µg/L, respectively) were detected in control wines (irrespective of O_3_ treatment), along with low levels (<25 µg/L) of VP glycosides (Table 3). As expected, grapevine exposure to smoke resulted in significantly higher concentrations of smoke taint marker compounds: both VPs (guaiacol and cresols in particular) and VP glycosides (Table 3, Tables S3 and S4). In a recent study involving the application of smoke to Cabernet Sauvignon vines [18], Szeto and colleagues reported the most abundant VP glycosides present in wines made from smoke-affected grapes were the glucose glucoside of syringol, the pentose glucoside of guaiacol, and to a lesser extent, the rutinosides of cresols and phenol. However, in the current study, pentose glucosides of guaiacol, cresols and phenol, and the glucose glucoside of syringol were most abundant (Table S3).

**Figure 1:**
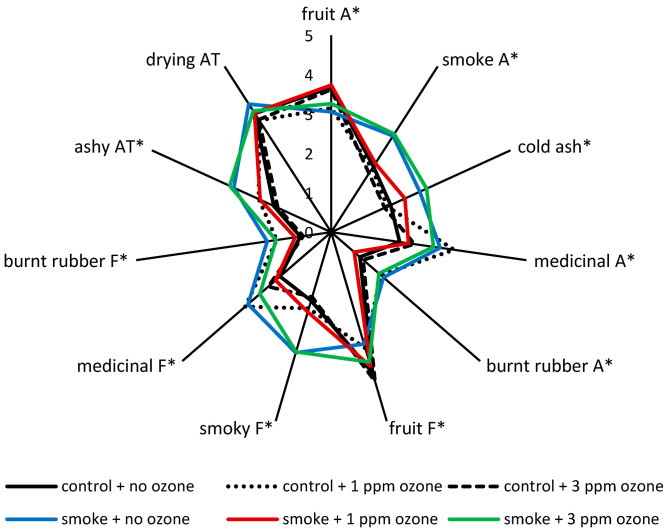
Sensory profiles of wines made from control and smoke-affected grapes, with and without post-harvest ozone treatment (at 1 ppm for 24 h or 3 ppm for 12 h); A = aroma; F = flavor; AT = aftertaste. Values are mean ratings of one blended wine per treatment, presented to 50 judges; * indicates statistical significance (*p* = 0.05, one-way ANOVA). Smoke exposure occurred at approximately 7 days post-veraison.

The 1 ppm O_3_ treatment of smoke-affected grapes not only resulted in significantly lower guaiacol and cresol levels in wine (i.e., 20–26% decreases), but also decreased wine VP glycosides by ~8–23% (Table 3). With the exception of a small (~15%) but statistically significant increase in the concentration of syringol glucose glucoside (Table S4), comparable VP and VP glycoside levels were observed for wines made from smoke-affected grapes with or without 3 ppm O_3_ treatment for 12 h (Table 3 and Table S3). These results provide further evidence that the 24 h post-harvest treatment of smoke-affected grapes with 1 ppm of O_3_ partially mitigated the effects of grapevine exposure to smoke, and showed good agreement with sensory results (Figure 1).

The sensory profiles of control wines were similar (Figure 1), irrespective of ozone treatment. With the exception of the control wine made from grapes that were treated with 1 ppm of O_3_, which for some reason exhibited enhanced medicinal attributes and therefore a loss in fruit intensity on the palate (Table S5), these wines were characterized by fruit aromas and flavors, and the absence of smoke-related sensory properties (Figure 1). In comparison, the wine made from smoke-affected grapes exhibited the distinctive smoky, cold ash, medicinal attributes and ashy aftertaste that have become synonymous with smoke taint (Figure 1). Wine made from smoke-affected grapes that were treated with 3 ppm of O_3_ for 12 h had a similar sensory profile; the only significant difference was a moderate increase in fruit flavor (Table S5). However, the wine made from smoke-affected grapes that were treated with 1 ppm of O_3_ for 24 h exhibited more intense fruit aroma and flavor, and importantly, significantly diminished smoke attributes, especially on the palate (Figure 1, Table S5).

Collectively, these results provide evidence that 24 h post-harvest treatment of smoke-affected grapes with 1 ppm of O_3_ partially mitigated the effects of grapevine exposure to smoke. Ozonation therefore offers a promising strategy for remediation of smoke taint, particularly if compositional and sensory outcomes can be improved with further optimization of O_3_ dose rate and/or treatment times.

### 2.3. Influence of Post-Harvest Smoke Exposure and O_3_ Treatment on Composition of Grapes

#### 2.3.1. VPs and VP Glycosides

**Figure 2:**
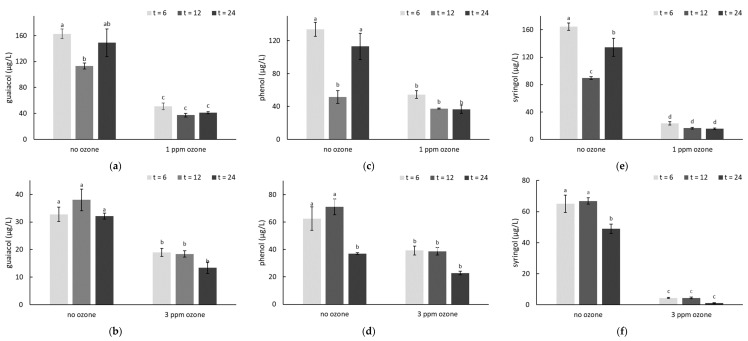
Concentrations (µg/L) of (**a**,**b**) guaiacol, (**c**,**d**) phenol and (**e**,**f**) syringol in smoke-affected grapes, with and without ozone treatment (at (**a**,**c**,**e**) 1 or (**b**,**d**,**f**) 3 ppm for 6, 12 or 24 h).Values are means of three replicates (*n* = 3). Different letters indicate statistical significance (*p* = 0.05, one-way ANOVA). Post-harvest smoke treatments were applied on consecutive days, but smoke density was lower on the second day of treatment due to increased wind.

Numerous studies have demonstrated that following their uptake from smoke into grapes, VPs accumulate in glycoconjugate forms due to rapid in vivo glycosylation [8,18,43,60,61]. As such, when post-harvest O_3_ treatments were applied in the first trial, smoke-derived VPs were only present in glycoconjugate forms (Table 1 and Table S1). Additional experiments involving post-harvest applications of smoke and O_3_ were therefore undertaken (over two consecutive days) to determine the effect of ozonation on free (aglycone) VPs. Smoke exposure of excised Merlot bunches was carried out in a purpose-built smoke chamber, after which 24 h O_3_ treatments commenced, with fruit exposed to smoke on the first and second days treated with 1 and 3 ppm O_3_, respectively. However, it should be noted that due to windy conditions being experienced on the second day of smoke application, fruit was exposed to less dense smoke, such that VPs were higher in grapes treated with 1 ppm of O_3_, than with 3 ppm of O_3_ (Figure 2, Table S6). Elevated concentrations of VPs were nevertheless detected in grapes as a consequence of post-harvest smoke exposure; guaiacol and syringol were the most abundant VPs present, and 4-methylsyringol and 4-methylguaiacol the least abundant, in agreement with previous research [11,14,18,62]. 

After 24 h of O_3_ treatment, smoke-affected grapes had significantly lower VP concentrations, irrespective of dose rate (Figure 2, Table S6). Volatile phenol losses were typically achieved within 6 h of O_3_ treatment, with similar VP levels observed between the three sampling times (i.e., t = 6, 12 and 24 h); only *m*- and *p*-cresol concentrations appeared to decrease significantly after 24 h of 3 ppm O_3_ treatment (relative to concentrations observed at t = 6; Table S6). Losses (relative to the corresponding smoke no O_3_ treatment, at t = 6) ranged from ~40 to 100%, but losses for the more abundant smoke taint markers (i.e., guaiacol, phenol, cresols and syringol) were typically ≥ 70% and ≥ 55% after 24 h O_3_ treatments at 1 and 3 ppm, respectively. Variation was also observed amongst the VP concentrations of smoke-affected grapes that were not treated with O_3_ (Figure 2), which might reflect variation in berry weight, smoke exposure and possibly even some degree of glycosylation, which has been shown to occur following smoke exposure of excised bunches [63].

These results suggest the mitigating effects of ozonation were more effective when smoke-derived VPs were present in free (aglycone) form, rather than glycoconjugate forms. This finding was consistent with previous research that reported bound volatile compounds were less sensitive to O_3_ treatment than free volatile compounds [39]. The observed loss of VPs is likely attributable to oxidation, either directly or via the formation of intermediate hydroxyl radicals, as previously hypothesized [35]. The current study did not attempt to isolate and identify the byproducts of O_3_ treatment (in either trial), but this would constitute worthwhile future research, in order to better understand the consequences of ozonation of smoke-affected grapes, and might help to explain the contrasting results observed for different O_3_ dose rates in Trial 1.

#### 2.3.2. Anthocyanins and Tannins

**Figure 3:**
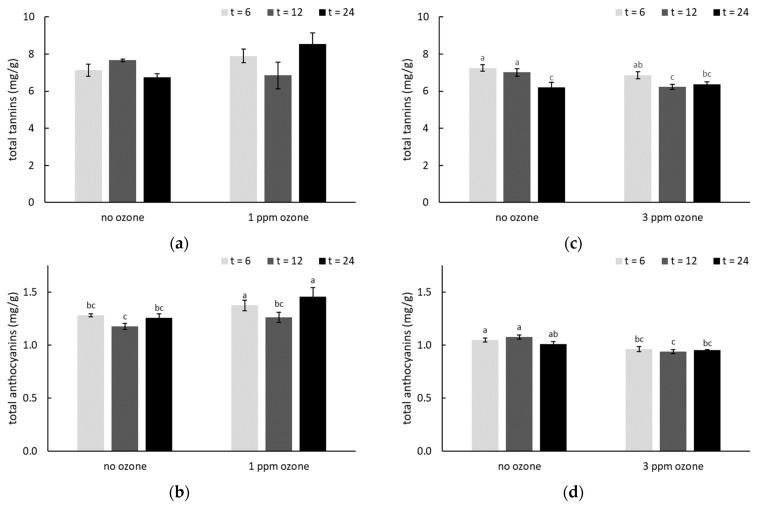
Total tannin and anthocyanin concentrations (mg/g) in smoke-affected grapes, with and without post-harvest ozone treatment (at (**a**,**b**) 1 or (**c**,**d**) 3 ppm for 6, 12 or 24 h).Values are means of three replicates (*n* = 3). Different letters indicate statistical significance (*p* = 0.05, one-way ANOVA); ns = not significant. Post-harvest smoke treatments were applied on consecutive days, but smoke density was lower on the second day of treatment due to increased wind.

Ozone treatment has previously been shown to simultaneously induce oxidation of grape volatiles and biosynthesis of secondary metabolites, including phenolic compounds [44,64]. The total tannin and anthocyanin concentrations of smoke-affected grapes with and without O_3_ treatment were therefore compared (Figure 3, Table S7). After 24 h of treatment with 1 ppm of O_3_, significantly higher tannin and anthocyanin concentrations were observed compared with smoke-affected grapes which were not ozonated (Figure 3a,b). In contrast, where smoke-affected grapes were treated with 3 ppm of O_3_, significant differences were only observed after 12 h of treatment (Figure 3c,d), and tannin and anthocyanin concentrations both decreased. Again, the different responses observed between O_3_ dose rates were not unexpected. As a strong oxidant, there is potential for O_3_ to oxidize polyphenols, rather than stimulate their production, depending on duration of treatment [65]. At appropriate doses, O_3_ can induce defense mechanisms that extend the postharvest life of fruit, but excessive ozonation can cause injury to fruit [66]. In addition, the different responses to ozone on fruits might also be due to the species specificity, treatment methods, storage condition, etc. Increased levels of anthocyanins and tannins were observed in table grapes following overnight (12 h) O_3_ treatment [28] and it has previously been established that exposure to 1 ppm O_3_ is sufficient to promote polyphenol synthesis in grapes [29,32,44,45,59]. In the current study, 3 ppm O_3_ treatment did not achieve the same outcome, and despite an apparent treatment effect after 12 h, there were no significant differences after 24 h of ozonation at 3 ppm (Figure 3c,d, Table S7), mirroring the results obtained for VP glycosides.

## 3. Materials and Methods

### 3.1. Smoke Treatment of Grapevines and Post-Harvest Ozone Treatment of Grapes (Trial 1)

Merlot grapevines growing in a vineyard located at the University of Adelaide’s Waite Campus in Urrbrae, South Australia (34°58′ S, 138°38′ E) were exposed to smoke for 1 h (at approximately 7 days post-veraison) during the 2019/2020 growing season, using a purpose-built smoke tent (2.0 × 6.0 × 2.5 m) and commercial fire box smokers, as previously described [18]. Grapevines were planted (in 1992) in north–south aligned rows, on their own roots and trained to a bilateral cordon, with a vertical shoot positioned trellis system, hand-pruned to a two-node spur system, and drip irrigated twice weekly from fruit set to pre-harvest. Smoke treatments were applied to a total of nine adjacent vines, with a panel of three vines enclosed in the smoke tent for each of three replicate applications of smoke. To maintain smoke production throughout the duration of each treatment, fuel (~2.4 kg of barley straw per smoke application) was combusted incrementally (i.e., ~200 g every 5 min). Smoke-affected grapevines were harvested when total soluble solids (TSS) reached 24 °Brix (approximately 4 weeks after smoke exposure). Fruit from nine control grapevines (from three adjacent panels, separated from smoke-affected grapevines by a panel of buffer vines) was harvested at the same time and level of TSS. Alternating bunches were hand-picked from control and smoke-affected grapevines on each of two consecutive days, to allow two different post-harvest ozone treatments. Berry samples (30 berries per replicate, per treatment, chosen randomly) were collected, homogenized (T18 Ultra Turrax, IKA, Staufen, Germany) and frozen at −4 °C for quantification of VPs and VP glycoconjugates (approximately five months after sampling).

Following the first harvest, control and smoke-affected bunches of Merlot grapes (approximately 12 kg per treatment) were each randomly divided into six parcels of fruit (~2 kg each). Three parcels of control fruit and three parcels of smoke-affected fruit were treated with 1 ppm of gaseous ozone (produced with an A series ozone generator, PC Engineering, Uggiate-Trevano, Italy) for 24 h in a 4 °C cold room. Dose rates were chosen based on previous studies involving ozone treatment of grapes [28,30,44], which suggested a minimal effect when ozone was applied at 1 ppm for only 12 h.

Berry samples (30 berries per replicate, per treatment) were collected (at t = 6, t = 12 and t = 24 h) for chemical analysis. The remaining parcels of control and smoke-affected fruit were not treated with ozone but were stored in the 4 °C cold room for the duration of ozone treatment (i.e., as “control no ozone” and “smoke no ozone” treatments). Fruit from the second harvest was divided into three parcels of control fruit and three parcels of smoke-affected fruit (~2 kg per parcel, per treatment), and treated with 3 ppm of gaseous ozone for 12 h. Berry samples were again collected (as above, at t = 6 and t = 12 h) for chemical analysis. On completion of ozone treatments (24 h for 1 ppm O_3_; 12 h for 3 ppm O_3_) the remaining fruit was used for small-scale winemaking, with replicate fruit parcels from each treatment becoming wine replicates.

### 3.2. Post-Harvest Smoke and Ozone Treatment of Grapes (Trial 2)

Grape bunches (approximately 2.5 kg) were harvested from unsmoked Merlot grapevines (from panels adjacent to the control grapevines described in Section 3.1), on each of two consecutive days (commencing two days after grapes for trial 1 were harvested). Following each harvest, fruit was exposed to smoke for 30 min, using a purpose-built smoke chamber (0.8 × 0.8 × 1.5 m) and a commercial fire box smoker. Grape bunches were suspended on wire frames and fuel (~200 g of barley straw) combusted to produce smoke, which was blown into the chamber via an aluminum foil flexible duct (~3.5 m × 150 mm) using an air pump. However, due to windy conditions being experienced on the second day of smoke application, the density of smoke differed between the two smoke treatments. After smoke exposure, fruit was randomly divided into six parcels of fruit (~400 g per parcel). Three parcels of smoke-affected fruit were treated with gaseous ozone for 24 h in a 4 °C cold room, at 1 and 3 ppm for fruit harvested on the first and second days of trial 2, respectively. The remaining parcels of smoke-affected fruit (from each harvest) were not treated with ozone but were stored in the 4 °C cold room for the duration of ozone treatment (i.e., as “smoke no ozone” treatments). Berry samples (30 berries per replicate, per treatment) were collected (at t = 6, t = 12 and t = 24 h) for chemical analysis.

### 3.3. Winemaking

Grape bunches (~2 kg per replicate, per treatment) were crushed and de-stemmed, with the addition of 50 mg/kg sulfur dioxide (added as an 8% solution of potassium metabisulphite). The pH of must was adjusted to 3.5 with the addition of tartaric acid, prior to inoculation with 150 mg/L of PDM yeast (Maurivin, AB Biotek, Sydney, NSW, Australia) and addition of diammonium phosphate (100 mg/L). Musts were fermented on skins at ambient temperature (25–27 °C) for one week, with the cap plunged twice daily. When wines approached dryness (i.e., ~2 g/L residual sugar), they were pressed and held at 25 °C until completion of fermentation (i.e., until residual sugars were <1 g/L), after which they were racked from gross lees and cold stabilized at 0 °C for 4 weeks. Wines did not undergo malolactic fermentation. Wine pH and free SO_2_ were adjusted to 3.5 and 20 mg/L, respectively, before bottling (in 375 mL glass bottles, with screw cap closures). Bottles were stored at 15 °C for two months prior to sensory analysis. Prior to bottling, wines were sampled for chemical analysis.

### 3.4. Chemical Analysis of Grapes and Wine

#### 3.4.1. Determination of Volatile Phenols

The concentrations of VPs (guaiacol, 4-methylguaiacol, phenol, *o*-, *m*- and *p*-cresol, syringol and 4-methylsyringol) were measured in grape homogenate and wine (three replicates each), using gas chromatography–mass spectrometry (GC-MS) and stable isotope dilution analysis (SIDA) methods described previously [41,67]. These publications describe the preparation of isotopically labeled standards (d_4_-guaiacol and d_3_-syringol for analysis of grape juices performed at the University of Adelaide and *d*_3_-guaiacol, *d*_3_-4-methylguaiacol, *d*_7_-*o*-cresol and *d*_3_-syringol for analysis of wine performed by the Australian Wine Research Institute’s (AWRI) Commercial Services Laboratory), as well as method validation and instrumental operating conditions. All measurements were performed using an Agilent 6890 gas chromatograph coupled to a 5973 mass spectrometer (Agilent Technologies, Forest Hill, Vic., Australia). The limit of quantitation for VPs was 1–2 µg/L.

#### 3.4.2. Determination of Volatile Phenol Glycoconjugates

The concentrations of VP glycosides were measured in grape homogenate and wine (three replicates each), as syringol glucose-glucoside (gentiobioside) equivalents, using liquid chromatography–tandem mass spectrometry (HPLC-MS/MS) and previously published SIDA methods [41,42]. These publications describe the preparation of isotopically labeled standards (*d*_3_-syringol gentiobioside), as well as method validation and instrumental operating conditions. Measurements were performed on an Agilent 1200 high-performance liquid chromatograph (HPLC) equipped with a 1290 binary pump, coupled to an AB SCIEX Triple Quad^TM^ 4500 tandem mass spectrometer, with a Turbo V^TM^ ion source (Framingham, MA, USA). Data acquisition and processing were performed using Analyst software (version 1.7 AB SCIEX). The limit of quantitation for VP glycosides was 1 µg/kg (as syringol glucose-glucoside equivalents).

#### 3.4.3. Determination of Total Tannins and Anthocyanins

Total tannins and anthocyanins were determined in grape homogenate (three replicates) using the methyl cellulose precipitable (MCP) tannin assay and high-performance liquid chromatography (HPLC) [68], respectively. Anthocyanin profiles were measured using an Agilent 1100 HPLC (Agilent Technologies, Waldbronn, Germany) equipped with a quaternary pump and diode array detector. Separation was achieved with a Synergi Hydro-6q column (150 × 2 mm, 4 mm, 80 Å) operating at 25 °C and protected by a guard column (4 × 2 mm) of the same material (Phenomenex, Lane Cove, NSW, Australia). Solvents were the same as those reported previously [68]: (A) 1% acetonitrile, 1.5% phosphoric acid in water; and (B) 20% solvent A, 80% acetonitrile for gradient elution at a flow rate of 0.4 mL/min: 0 min (14.5% solvent B), 18 min (27.5% solvent B), 24 min (27.5% solvent B), 25 min (50.0% solvent B), 26 min (50.0% solvent B), 30 min (100% solvent B), 32 min (100% solvent B), 32.01 min (14.5% solvent B), and 40 min (14.5% solvent B). The pump seal wash solution was 10% isopropanol in water. A 20 µL injection volume was used for each sample and signals were recorded at an absorbance of 520 nm. Data acquisition and processing were performed using Agilent ChemStation software (version A.09.03). Anthocyanins were quantified at 520 nm against an external calibration curve of malvidin-3-glucoside hydrochloride (Sigma-Aldrich Pty Ltd.; subsidiary of Merck, North Ryde, NSW, Australia) from 1 to 500 mg/L.

#### 3.4.4. Determination of Basic Wine Chemistry Parameters

Residual sugars were measured enzymatically (using a glucose/fructose enzymatic test kit from Vintessential Laboratories Pty. Ltd., Dromana, VIC, Australia) using a Chemwell 2910 automated analyzer (Awareness Technology Inc., Palm City, FL, USA). pH and titratable acidity (TA, expressed as g/L tartaric acid) were measured using a Mettler Toledo T50 autotitrator coupled to a Mettler Toledo InMotion Flex autosampler (Port Melbourne, VIC, Australia). Ethanol content (% alcohol by volume, abv) was measured with an alcolyzer (Anton Paar, Graz, Austria). Wine color density, wine hue and total phenolics were determined by the modified Somers color assay [68] using an Infinite^®^ 200 PRO spectrophotometer (Tecan, Männedorf, Switzerland). Chemical analyses were performed on each of the three wine replicates.

### 3.5. Sensory Analysis of Wines

Wines from each experimental treatment were assessed by wine sensory experts from the University of Adelaide for evidence of faults or differences between replicates, before replicates were blended. The sensory profiles of wines (one blended wine per treatment) were then determined using the rate-all-that-apply (RATA) method [69] and a panel comprising staff and students from the University of Adelaide and the Australian Wine Research Institute, and regular wine consumers (*n* = 50, 12 males and 38 females, aged between 20 and 74 years). Prior to wine evaluation, panelists completed a brief induction, during which they were familiarized with both the RATA procedure and a list of attributes and their definitions (Table S8), which were adapted from previous studies [11,70]. RATA assessments were conducted in sensory booths at 22–23 °C under sodium lights, with wine aliquots (30 mL) presented monadically, in a randomized order, in covered, 3-digit coded 215 mL stemmed International Organization for Standardization wine glasses. During assessment, panelists determined the attributes they perceived to be applicable to each wine sample and rated the intensity of attributes using line scales (where 0 = “not perceived”, 1 = “extremely low” and 9 = “extremely high”). Panelists rinsed thoroughly with water and rested for at least 1 min between samples, with plain crackers provided as palate cleansers. Data were acquired with Red Jade software (Redwood Shores, CA, USA).

### 3.6. Statistical Analysis

Chemical data were analyzed by analysis of variance (ANOVA) using GenStat (19th Edition, VSN International Limited, Herts, UK). Mean comparisons were performed by least significant difference (LSD) multiple comparison test at *p* < 0.05. Sensory data were analyzed using SenPAQ (version 5.01, Qi Statistics, Reading, UK) and XLSTAT (version 2018.1.1, Addinsoft, NY, USA). Mean comparisons were performed by Fisher’s least significant difference (LSD) multiple comparison test at *p* < 0.05.

## 4. Conclusions

Smoke taint remains a significant issue of concern for the global wine industry and improved strategies are needed to mitigate the negative effects of grapevine smoke exposure on the composition and sensory properties of grapes, and therefore wine. This study demonstrated the potential for post-harvest O_3_ treatment to be used to mitigate the intensity of taint perceived in wine made from smoke-affected grapes. Differences were observed between the concentration of VPs (in both free and glycoconjugate forms) in smoke-affected grapes with and without O_3_ treatment. At lower dose rates (i.e., at 1 ppm) ozonation gave significantly lower VP glycoside concentrations, which resulted in a perceivable improvement in wine sensory properties, i.e., less apparent smoky, medicinal, burnt rubber and ashy characters. The O_3_ dose rate may, however, influence the efficacy of treatment, such that higher doses induce cellular defense mechanisms to oxidative stress that negate or hinder any mitigation of smoke taint.

Furthermore, the timing of O_3_ treatment after grapevine smoke exposure may be important, with results suggesting free VPs are more susceptible to the effects of O_3_ (presumably oxidation) than their glycosylated forms. Further research is needed to optimize the timing, dose rate and duration of O_3_ treatment of smoke-affected grapes, and to fully understand both the chemical and biochemical consequences of ozonation (including any potential impact on wine color and mouthfeel properties due to the effects of O_3_ on biosynthesis of phenolic compounds). However, collectively, the results presented here demonstrate the efficacy of post-harvest ozone treatment as a promising new strategy for mitigation of smoke taint in wine.

## Figures and Tables

**Table 1:** Concentration (µg/kg) of volatile phenol glycosides in control and smoke-affected grapes, with and without post-harvest ozone treatment (at 1 ppm for 24 h or 3 ppm for 12 h).

Treatment		Guaiacol Glycosides	4-Methyl Guaiacol Glycosides	Phenol Glycosides	Cresol Glycosides	Syringol Glycosides	4-Methyl Syringol Glycosides
control no O_3_		7.6 ± 0.3 d	4.7 ± 0.8 d	19 ± 2.3 e	20 ± 1.9 d	4.4 ± 0.2 e	1.8 ± 0.3 d
control 1 ppm O_3_	t = 6	12 ± 1.4 d	5.0 ± 0.2 d	24 ± 2.5 e	23 ± 1.8 d	8.9 ± 1.3 e	1.8 ± 0.1 d
	t = 12	12 ± 0.6 d	5.3 ± 0.6 d	24 ± 0.9 e	24 ± 1.4 d	8.3 ± 0.6 e	1.8 ± 0.2 d
	t = 24	9.9 ± 1.3 d	4.7 ± 0.6 d	22 ± 4.1 e	22 ± 3.1 d	6.8 ± 1.3 e	1.8 ± 0.5 d
control 3 ppm O_3_	t = 6	8.1 ± 0.5 d	4.7 ± 0.8 d	17 ± 1.4 e	21 ± 2.4 d	5.2 ± 0.5 e	1.7 ± 0.3 d
	t = 12	7.4 ± 0.7 d	4.3 ± 0.5 d	17 ± 1.8 e	18 ± 2.2 d	4.0 ± 0.3 e	1.6 ± 0.2 d
smoke no O_3_		252 ± 14 b	51 ± 5.9 ab	227 ± 18 bc	261 ± 23 b	300 ± 22 bc	19 ± 0.5 ab
smoke 1 ppm O_3_	t = 6	231 ± 8.0 b	45 ± 3.6 bc	235 ± 27 bc	257 ± 33 b	316 ± 24 b	18 ± 0.9 bc
	t = 12	246 ± 4.3 b	47 ± 4.3 b	245 ± 3.7 b	261 ± 21 b	327 ± 27 ab	19 ± 2.2 ab
	t = 24	207 ± 35 c	41 ± 6.9 c	189 ± 29 d	208 ± 30 c	265 ± 33 d	16 ± 2.2 c
smoke 3 ppm O_3_	t = 6	291 ± 12 a	57 ± 3.9 a	282 ± 25 a	308 ± 25 a	348 ± 7.8 a	21 ± 2.0 a
	t = 12	230 ± 26 bc	47 ± 3.9 b	216 ± 23 cd	238 ± 24 bc	282 ± 32 cd	18 ± 2.1 bc
	*p*	<0.001	<0.001	<0.001	<0.001	<0.001	<0.001

Values are means of three replicates (*n* = 3) ± standard deviation measured as syringol glucose-glucoside equivalents. Different letters (within columns) indicate statistical significance (*p* = 0.05, one way ANOVA). Smoke exposure occurred at approximately 7 days post-veraison.

**Table 2:** Basic composition of wines made from control and smoke-affected grapes, with and without post-harvest ozone treatment (at 1 ppm for 24 h or 3 ppm for 12 h).

**Treatment**		pH	TA (g/L)	Alcohol (% abv)	Wine Color Density (au)	Wine Color Hue	% Ionized Anthocyanins	SO_2_ Resistant Pigments (au)	Total Phenolics (au)
control	no O_3_	3.9 ± 0.01 a	6.1 ± 0.04	14.5 ± 0.1 ab	7.1 ± 0.3 a	0.69 ± 0.01 a	20.2 ± 0.3	1.9 ± 0.06 b	30.7 ± 1.7 a
	1 ppm O_3_	3.8 ± 0.02 ab	7.0 ± 0.10	14.2 ± 0.2 c	7.0 ± 0.2 a	0.64 ± 0.01 c	22.3 ± 2.1	2.3 ± 0.13 a	26.1 ± 0.1 b
	3 ppm O_3_	3.8 ± 0.01 ab	5.8 ± 0.34	14.8 ± 0.1 a	6.2 ± 0.2 b	0.71 ± 0.01 a	18.1 ± 0.8	1.8 ± 0.08 b	26.1 ± 0.6 b
smoked	no O_3_	3.7 ± 0.13 b	6.6 ± 0.28	14.0 ± 0.1 c	6.6 ± 0.3 ab	0.67 ± 0.0 b	22.3 ± 1.5	1.9 ± 0.05 b	29.4 ± 0.6 a
	1 ppm O_3_	3.7 ± 0.04 b	7.5 ± 0.67	14.4 ± 0.1 ab	6.7 ± 0.2 ab	0.63 ± 0.01 c	20.0 ± 1.5	1.8 ± 0.04 b	27.3 ± 0.8 b
	3 ppm O_3_	3.7 ± 0.03 b	7.1 ± 0.63	14.1 ± 0.1 c	6.6 ± 0.1 ab	0.64 ± 0.0 bc	20.8 ± 0.2	1.8 ± 0.05 b	27.0 ± 0.4 b
	*p*	0.011	ns	<0.001	0.039	<0.001	ns	<0.001	0.002

Values are means of three replicates (*n* = 3) ± standard deviation. Different letters (within columns) indicate statistical significance (*p* = 0.05, one way ANOVA); ns = not significant. Smoke exposure occurred at approximately 7 days post-veraison.

**Table 3:** Concentration (µg/L) of volatile phenols and volatile phenol glycosides in wines made from control and smoke-affected grapes, with and without post-harvest ozone treatment (at 1 ppm for 24 h or 3 ppm for 12 h).

Treatment		Guaiacol	4-Methyl Guaiacol	Cresols	Syringol	Guaiacol Glycosides	4-Methyl Guaiacol Glycosides	Phenol Glycosides	Cresol Glycosides	Syringol Glycosides	4-Methyl Syringol Glycosides
control	no O_3_	1.0 ± 0.0 c	nd	nd	3.0 ± 0.0 b	9.4 ± 0.2 c	4.8 ± 0.6 c	19 ± 0.9 c	20 ± 1.3 c	3.4 ± 0.2 c	1.0 ± 0.1 b
	1 ppm O_3_	1.0 ± 0.0 c	nd	nd	2.7 ± 0.6 b	13 ± 2.4 c	5.3 ± 0.8 c	23 ± 2.0 c	23 ± 3.9 c	6.1 ± 2.5 c	1.0 ± 0.4 b
	3 ppm O_3_	1.0 ± 0.0 c	nd	nd	3.0 ± 0.0 b	8.8 ± 0.6 c	4.4 ± 0.4 c	21 ± 1.2 c	18 ± 1.1 c	3.7 ± 0.3 c	tr
smoke	no O_3_	15 ± 1.5 a	1.0 ± 0.0	7.7 ± 1.5 a	4.7 ± 0.3 a	295 ± 23 a	59 ± 4.4 a	249 ± 15 a	280 ± 5.2 a	240 ± 10 ab	13 ± 0.9 a
	1 ppm O_3_	12 ± 2.1 b	nd	5.7 ± 1.5 b	4.0 ± 0.0 a	232 ± 15 b	47 ± 2.4 b	198 ± 15 b	217 ± 11 b	213 ± 12 b	12 ± 0.7 a
	3 ppm O_3_	14 ± 2.3 ab	nd	7.4 ± 1.2 a	4.3 ± 0.3 a	329 ± 57 a	62 ± 11 a	273 ± 51 a	314 ± 63 a	273 ± 42 a	13 ± 3.6 a
	*p*	< 0.001	–	<0.001	< 0.001	<0.001	<0.001	<0.001	<0.001	<0.001	<0.001

Values are means of three replicates (*n* = 3) ± standard deviation measured as syringol glucose-glucoside equivalents; nd = not detected; tr = trace (i.e., 0.5–1 µg/kg). Different letters (within columns) indicate statistical significance (*p* = 0.05, one way ANOVA); ns = not significant. Smoke exposure occurred at approximately 7 days post-veraison.

## Data Availability

The data presented in this study are available on request from the corresponding author (pending privacy and ethical considerations).

## Supplementary Materials

The following are available online. Table S1: Concentration (µg/kg) of volatile phenol glycoconjugates in control and smoke-affected grapes, with and without post-harvest ozone treatment; Table S2: Concentration (mg/g) of anthocyanins in control and smoke-affected grapes, with and without post-harvest ozone treatment; Table S3: Concentration (µg/L) of volatile phenol glycoconjugates in wines made from control and smoke-affected grapes, with and without post-harvest ozone treatment (at 1 ppm for 24 h or 3 ppm for 12 h); Table S4: Concentration (µg/L) of volatile phenols in wines made from control and smoke-affected grapes, with and without post-harvest ozone treatment (at 1 ppm for 24 h or 3 ppm for 12 h); Table S5: Mean intensity ratings for sensory attributes of wines made from control and smoke-affected grapes, with and without post-harvest ozone treatment (at 1 ppm for 24 h or 3 ppm for 12 h); Table S6: Concentration (µg/L) of volatile phenols in juice from smoke-affected grapes, with and without post-harvest ozone treatment (at 1 or 3 ppm for 6, 12 or 24 h); Table S7: Concentration (mg/g) of anthocyanins in control and smoke-affected grapes, with and without post-harvest ozone treatment; Table S8: Aroma and palate attributes used in sensory analysis of wines.

## Sample Availability

Samples of the compounds are not available from the authors.
