# Supplementary material for: Potential Mitigation of Smoke Taint in Wines by Post-Harvest Ozone Treatment of Grapes
Source: Molecules. 2021 Mar 23;26(6):1798. doi: 10.3390/molecules26061798 (PMC8004780; doi:10.3390/molecules26061798)
Supplement: Supplementary file 1 [file molecules-26-01798-s001.pdf]

molecules

MDPI

Supplementary Materials

Article

Potential Mitigation of Smoke Taint in Wines by Post-Harvest Ozone Treatment of Grapes

Margherita Modesti [1,2,*], Colleen Szeto [2,3], Renata Ristic [2,3], WenWen Jiang [4], Julie Culbert [4], Keren Bindon [4], Cesare Catelli[5], Fabio Mencarelli[6] , Pietro Tonutti [1], Kerry Wilkinson [2,3]

[1] Life Sciences Institute, Scuola Superiore Sant'Anna, Piazza Martiri della libertà 33, 5612, Pisa, Italy; margherita.modesti@santannapisa.it (M.M.); pietro.tonutti@santannapisa.it (P.T.)

[2] Department of Wine Science, Waite Research Institute, The University of Adelaide, PMB 1, Glen Osmond, SA, 5064, Australia; colleen.szeto@adelaide.edu.au (C.S.); renata.ristic@adelaide.edu.au (R.R.); kerry.wilkinson@adelaide.edu.au (K.W.)

[3] The Australian Research Council Training Centre for Innovative Wine Production, PMB 1, Glen Osmond, SA, 5064, Australia

[4] The Australian Wine Research Institute, PO Box 197, Glen Osmond, SA, 5064, Australia; julie.culbert@awri.com.au (J.C.); maddy.jiang@awri.com.au (W.J.); keren.bindon@awri.com.au (K.B.)

[5] PC Engineering srl, VIA Roma 81, 22019 , Uggiate Trevano (CO), Italy; cesare.catelli@pinco-sa.com (C.C.)

[6] DAFE, University of Pisa, Via del Borghetto 80, 56124 Pisa, Italy; fabio.mencarelli@unipi.it (F.M.)

* Correspondence: margherita.modesti@santannapisa.it

Table S1. Concentration (μg/kg) of volatile phenol glycoconjugates in control and smoke-affected grapes, with and without post-harvest ozone treatment.

Treatment		guaiacol glycosides				4-methylguaiacol glycosides			phenol glycosides				cresol glycosides				syringol glycosides			4-methylsyringol glycosides	
		G	GG	PG	R	GG	PG	R	G	GG	PG	R	G	GG	PG	R	G	GG	PG	GG	PG
control no O_3		nd	tr	5.9 d	tr	nd	2.5 d	1.9 e	1.1	nd	17 e	tr	nd	1.1	17 d	1.9 e	tr	1.6 e	1.9 e	tr	1.2
control 1 ppm O_3	t = 6	nd	1.3 d	9.3 d	1.0 d	nd	2.9 d	1.9 e	nd	nd	22 e	1.0 e	nd	tr	19 d	2.6 e	tr	5.9 e	2.2 e	tr	1.0
	t = 12	nd	1.5 d	9.3 d	1.1 d	nd	3.1 d	1.9 e	tr	nd	22 e	1.1 e	nd	1.0	20 d	2.7 e	tr	5.6 e	2.1 e	tr	1.1
	t = 24	nd	1.1 d	7.6 d	1.2 d	nd	2.7 d	1.8 e	tr	nd	20 e	1.0 e	tr	1.1	18 d	2.2 e	1.2	3.6 e	2.1 e	tr	1.0
control 3 ppm O_3	t = 6	nd	1.0 d	5.9 d	1.2 d	nd	2.5 d	1.9 e	nd	nd	16 e	tr	nd	1.2	18 d	1.8 e	tr	2.5 e	2.0 e	tr	1.0
	t = 12	nd	tr	5.4 d	1.1 d	nd	2.3 d	1.8 e	tr	nd	16 e	tr	nd	1.0	15 d	1.7 e	tr	1.5 e	1.8 e	tr	1.0
smoke no O_3		3.3	66 b	162 b	20 ab	6.3	27 ab	17 ab	2.6	2.9	192 bc	29 b	5.3	1.6	194 b	60 ab	3.2	276 bc	21 cd	15 ab	3.9
smoke 1 ppm O_3	t = 6	2.9	59 c	153 b	17 c	5.7	25 bc	14 cd	2.2	3.1	205 bc	24 cd	5.2	1.7	198 b	52 c	3.0	290 b	23 ab	14 b	4.0
	t = 12	2.9	68 b	158 b	18 bc	6.5	26 b	15 cd	2.2	3.2	212 b	27 bc	4.8	1.6	200 b	55 bc	3.6	301 ab	22 bc	15 sb	3.7
	t = 24	2.8	53 c	136 c	16 c	5.3	22 c	13 d	1.8	2.6	163 d	22 d	4.0	1.3	159 c	43 d	2.8	243 d	20 d	12 c	3.6
smoke 3 ppm O_3	t = 6	3.6	76 a	190 a	22 a	7.3	31 a	19 a	2.6	3.5	243 a	33 a	5.2	1.7	235 a	66 a	3.4	320 a	25 a	16 a	4.4
	t = 12	3.5	58 c	150 bc	18 bc	6.1	26 bc	16 bc	2.2	2.8	185 cd	26 bcd	4.6	1.6	179 bc	52 c	2.9	260 cd	19 d	15 ab	3.5
	p	–	<0.001	<0.001	<0.001	–	<0.001	<0.001	–	–	<0.001	<0.001	–	–	<0.001	<0.001	–	<0.001	<0.001	<0.001	–

Values are means of three replicates (n = 3) measured as syringol glucose-glucoside equivalents; nd = not detected; tr = trace (i.e. 0.5–1 μg/kg). Different letters (within columns) indicate statistical significance (p = 0.05, one way ANOVA). G = glucoside; GG = glucose-glucoside; PG = pentose-glucoside; R = rutinoside. Smoke exposure occurred at approximately 7 days post-véraison.

Table S2. Concentration (mg/g) of anthocyanins in control and smoke-affected grapes, with and without post-harvest ozone treatment.

Treatment		Del-3-*O*-gl	Cya-3-*O*-glc	Pet-3-*O*-glc	Peo-3-*O*-glc	Mal-3-*O*-glc	Del-acet-glc	Pet-acet-glc	Peo-acet-glc	Mal-acet-glc	Del-coum-glc	Cya-coum-glc & Mal-caff-glc	Pet-coum-glc	Peo-coum-glc	Mal-3-coum glc
control no O_3		0.13 ± 0.2	0.07 ± 0.1	0.11 ± 0.1	0.15 ± 0.02	0.41 ± 0.03	0.02 ± 0.0	0.03 ± 0.0	0.03 ± 0.0	0.12 ± 0.01	0.02 ± 0.0	0.01 ± 0.0	0.02 ± 0.0	0.03 ± 0.0	0.08 ± 0.0
control 1 ppm O_3	t = 6	0.11 ± 0.1	0.07 ± 0.0	0.10 ± 0.0	0.14 ± 0.0	0.35 ± 0.1	0.02 ± 0.0	0.02 ± 0.0	0.03 ± 0.0	0.10 ± 0.0	0.02 ± 0.0	0.01 ± 0.0	0.02 ± 0.0	0.03 ± 0.0	0.08 ± 0.0
	t = 12	0.13 ± 0.0	0.07 ± 0.0	0.10 ± 0.0	0.15 ± 0.1	0.37 ± 0.1	0.02 ± 0.0	0.03 ± 0.0	0.03 ± 0.0	0.10 ± 0.0	0.02 ± 0.0	0.02 ± 0.0	0.02 ± 0.0	0.04 ± 0.0	0.08 ± 0.0
	t = 24	0.11 ± 0.0	0.06 ± 0.0	0.10 ± 0.0	0.14 ± 0.0	0.35 ± 0.0	0.03 ± 0.0	0.03 ± 0.0	0.04 ± 0.0	0.12 ± 0.0	0.02 ± 0.0	0.02 ± 0.0	0.02 ± 0.0	0.03 ± 0.0	0.08 ± 0.0
control 3 ppm O_3	t = 6	0.11 ± 0.0	0.07 ± 0.0	0.10 ± 0.1	0.15 ± 0.1	0.36 ± 0.1	0.03 ± 0.0	0.03 ± 0.0	0.04 ± 0.0	0.12 ± 0.0	0.02 ± 0.0	0.02 ± 0.0	0.02 ± 0.0	0.04 ± 0.0	0.08 ± 0.0
	t = 12	0.10 ± 0.1	0.05 ± 0.1	0.09 ± 0.1	0.13 ± 0.1	0.35 ± 0.2	0.02 ± 0.0	0.03 ± 0.0	0.04 ± 0.0	0.11 ± 0.0	0.02 ± 0.0	0.01 ± 0.0	0.02 ± 0.0	0.03 ± 0.0	0.08 ± 0.0
smoke no O_3		0.14 ± 0.1	0.08 ± 0.0	0.11 ± 0.1	0.16 ± 0.1	0.39 ± 0.1	0.02 ± 0.0	0.03 ± 0.0	0.03 ± 0.0	0.10 ± 0.0	0.02 ± 0.0	0.02 ± 0.0	0.02 ± 0.0	0.04 ± 0.0	0.08 ± 0.1
smoke 1 ppm O_3	t = 6	0.14 ± 0.1	0.08 ± 0.0	0.11 ± 0.1	0.17 ± 0.1	0.41 ± 0.2	0.03 ± 0.0	0.03 ± 0.0	0.04 ± 0.0	0.11 ± 0.1	0.02 ± 0.0	0.02 ± 0.0	0.02 ± 0.0	0.04 ± 0.0	0.08 ± 0.1
	t = 12	0.13 ± 0.0	0.08 ± 0.0	0.11 ± 0.0	0.16 ± 0.1	0.39 ± 0.1	0.03 ± 0.0	0.03 ± 0.0	0.04 ± 0.0	0.11 ± 0.1	0.02 ± 0.0	0.02 ± 0.0	0.02 ± 0.0	0.04 ± 0.0	0.09 ± 0.0
	t = 24	0.12 ± 0.1	0.07 ± 0.1	0.10 ± 0.1	0.15 ± 0.1	0.40 ± 0.1	0.02 ± 0.0	0.03 ± 0.0	0.03 ± 0.0	0.11 ± 0.1	0.02 ± 0.0	0.02 ± 0.0	0.02 ± 0.0	0.04 ± 0.0	0.09 ± 0.0
smoke 3 ppm O_3	t = 6	0.12 ± 0.1	0.08 ± 0.1	0.10 ± 0.1	0.15 ± 0.1	0.36 ± 0.1	0.03 ± 0.0	0.03 ± 0.0	0.04 ± 0.0	0.11 ± 0.0	0.02 ± 0.0	0.02 ± 0.0	0.02 ± 0.0	0.04 ± 0.0	0.06 ± 0.0
	t = 12	0.14 ± 0.0	0.08 ± 0.1	0.11 ± 0.1	0.18 ± 0.1	0.42 ± 0.3	0.03 ± 0.0	0.03 ± 0.0	0.04 ± 0.0	0.12 ± 0.1	0.02 ± 0.0	0.02 ± 0.0	0.02 ± 0.0	0.04 ± 0.0	0.10 ± 0.1
	p	ns	ns	ns	ns	ns	ns	ns	ns	ns	ns	ns	ns	ns	ns

Values are means of three replicates (n = 3) ± standard deviation measured as malvidin 3-glucoside equivalents. Different letters (within columns) indicate statistical significance (p = 0.05, one way ANOVA); ns = not significant. Del = delphinidin; Cya= cyanidin; Pet= petunidin; Peo = peonidin; Mal – malvidin; glc = glucoside; acet = acetyl; coum = coumaroyl; caff = caffeoyl. Smoke exposure occurred at approximately 7 days post-véraison.

Table S3. Concentration (μg/L) of volatile phenol glycoconjugates in wines made from control and smoke-affected grapes, with and without post-harvest ozone treatment (at 1 ppm for 24 hours or 3 ppm for 12 hours).

Treatment		guaiacol glycosides				4-methylguaiacol glycosides			phenol glycosides				cresol glycosides				syringol glycosides			4-methylsyringol glycosides	
		**G**	**GG**	**PG**	**R**	**GG**	**PG**	**R**	**G**	**GG**	**PG**	**R**	**G**	**GG**	**PG**	**R**	**G**	**GG**	**PG**	**GG**	**PG**
control	no O_3	nd	nd	8 c	1.2 c	nd	3.1 c	1.6 c	2.5 c	nd	16 c	tr	tr	nd	16.5 c	2.3 c	nd	nd	3.5 c	nd	tr
	1 ppm O_3	nd	nd	11 c	1.8 c	nd	3.3 c	1.9 c	3.6 c	nd	18 c	1.4 c	1.0	nd	18.8 c	2.9 c	nd	2.0 c	3.7 c	nd	tr
	3 ppm O_3	nd	nd	7 c	1.5 c	nd	2.6 c	1.6 c	3.1 c	nd	16 c	1.1 c	tr	nd	15.0 c	2.2 c	nd	nd	3.5 c	nd	tr
smok	no O_3	16	4.1	239 a	36 a	1.1	34 a	23 a	30 a	2.7 a	177 a	38 a	10 b	nd	197 a	73 a	8.6 a	190 b	41 a	8.3	4.5
	1 ppm O_3	14	3.6	188 b	27 b	tr	28 b	18 b	24 b	2.3 b	142 b	30 b	7.5 c	nd	154 b	54 b	6.6 b	170 b	36 b	8.3	3.7
	3 ppm O_3	19	4.4	267 a	38 a	1.1	37 a	24 a	35 a	2.9 a	193 a	43 a	12 a	nd	222 a	81 a	9.3 a	219 a	45 a	9.0	4.2
p		ns	ns	<0.001	<0.001	–	<0.001	<0.001	<0.001	<0.001	<0.001	<0.001	<0.001	–	<0.001	<0.001	<0.001	<0.001	<0.001	ns	ns

Values are means of three replicates ($n = 3$) measured as syringol glucose-glucoside equivalents; nd = not detected; tr = trace (i.e. 0.5–1 μg/kg). Different letters (within columns) indicate statistical significance ($p = 0.05$, one way ANOVA); ns = not significant. G = glucoside; GG = glucose-glucoside; PG = pentose-glucoside; R = rutinoside. Smoke exposure occurred at approximately 7 days post-véraison.

Table S4. Concentration (μg/L) of volatile phenols in wines made from control and smoke-affected grapes, with and without post-harvest ozone treatment (at 1 ppm for 24 hours or 3 ppm for 12 hours).

**treatment**		**guaiacol**	**4-methyl guaiacol**	***o*-cresol**	***m*-cresol**	***p*-cresol**	**syringol**	**4-methyl-syringol**
control	no O_3	1 c	nd	nd	nd	nd	3.0 b	nd
	1 ppm O_3	1 c	nd	nd	nd	nd	2.7 b	nd
	3 ppm O_3	1 c	nd	nd	nd	nd	3.0 b	nd
smoked	no O_3	15 a	1	3.7 a	4.0	nd	4.7 a	nd
	1 ppm O_3	12 b	nd	2.7 b	3.0	nd	4.0 a	nd
	3 ppm O_3	14 ab	nd	3.7 a	3.7	nd	4.3 a	nd
	p	< 0.001	–	<0.001	ns	–	< 0.001	–

Values are means of three replicates (n = 3); nd = not detected (i.e. < 1 μg/L). Different letters (within columns) indicate statistical significance (p = 0.05, one way ANOVA); ns = not significant. Smoke exposure occurred at approximately 7 days post-véraison.

Table S5. Mean intensity ratings for sensory attributes of wines made from control and smoke-affected grapes, with and without post-harvest ozone treatment (at 1 ppm for 24 hours or 3 ppm for 12 hours).

**Attribute**	**Control**			**Smoke**			***p***
	**no O_3**	**1 ppm O_3**	**3 ppm O_3**	**no O_3**	**1 ppm O_3**	**3 ppm O_3**	
fruit A	3.7 ab	3.2 bc	3.6 abc	3.1 c	3.7 a	3.3 abc	0.001
smoke A	2.0 b	1.9 b	1.8 b	2.9 a	2.1 ab	3.0 a	<0.0001
cold ash A	1.7 b	1.6 b	1.5b	2.5 a	2.1 ab	2.7 a	<0.0001
earthy A	2.4	2.4	2.1	2.8	2.3	2.8	ns
medicinal A	1.8 c	3.2 a	2.1 bc	2.8 ab	2.0 bc	2.6 abc	<0.0001
burnt rubber A	1.0 bc	1.7 ab	1.1 abc	1.8 a	0.8c	1.6 ab	0.0001
metallic A	1.1 bcd	1.6 a	1.0 cd	1.4 abc	0.9 d	1.5 ab	0.011
fruit F	3.7 ab	3.1 cd	3.9 a	3.0 d	3.5 ab	3.4 bc	<0.0001
smoky F	1.8 b	2.0 b	1.7 b	3.2 a	2.1 b	3.2 a	<0.0001
burnt rubber F	0.8 bc	1.4 abc	0.7 c	1.6 a	0.9 bc	1.4 ab	0.0001
medicinal F	1.7 b	2.9 a	2.1ab	2.8 a	1.9 b	2.4 ab	0.0001
metallic F	1.2 ab	1.8 a	1.1 b	1.6 ab	1.2 ab	1.5 ab	0.035
ashy AT	1.6 b	2.0 ab	1.5 b	2.7 a	2.0 ab	2.8 a	<0.0001
woody AT	2.3	2	1.9	2.4	2.2	2.6	ns
acidity	3.8	3.9	3.8	3.8	3.8	3.8	ns
hotness	3.4	3.1	3.3	3.4	3.4	3.4	ns
bitterness	3.1	3.4	3.1	3.3	3.1	3.4	ns
astringency	3.5 ab	3.3 b	3.2 b	3.9 a	3.6 ab	3.7 ab	0.001
drying (AT)	3.6	3.4	3.4	3.9	3.6	3.7	ns

Values are means for one blended wine per treatment, presented to 50 judges. Different letters (within rows) indicate statistical significance (p = 0.05, one-way ANOVA); ns = not significant. A = aroma; F = flavor; AT = aftertaste. Smoke exposure occurred at approximately 7 days post-véraison.

Table S6. Concentration (µg/L) of volatile phenols in smoke-affected grapes, with and without post-harvest ozone treatment (at 1 or 3 ppm for 6, 12 or 24 hours).

Treatment		Guaiacol	4-Methyl Guaiacol	Phenol	*o*-Cresol	*m*-Cresol	*p*-Cresol	Syringol	4-Methyl Syringol
smoke no O_3	t = 6	163 a	29 a	134 a	63 a	53 a	11 a	165 a	13 a
	t = 12	113 b	18 b	51 b	47 b	32 b	8.1 b	90 c	7.3 c
	t = 24	149 ab	23 ab	113 a	59 ab	46 a	6.9 bc	134 b	9.8 b
smoke 1 ppm O_3	t = 6	51 c	10.7 c	55 b	22 c	18 c	4.5 cd	23 d	nd
	t = 12	37 c	8.7 c	37 b	19 c	15 c	4.8 cd	16 d	nd
	t = 24	41 c	8.4 c	37 b	20 c	14 c	3.4 d	16 d	nd
	p	< 0.0001	< 0.0001	< 0.0001	< 0.0001	< 0.0001	< 0.0001	< 0.0001	< 0.0001
smoke no O_3	t = 6	33 a	9.5 ab	63 a	22 a	21 ab	4.0 b	65 a	1.8
	t = 12	38 a	10.6 a	71 a	26 a	25 a	5.5 a	67 a	2.7
	t = 24	32 a	8.4 b	37 b	22 a	17 bc	2.5 cd	49 b	2.0
smoke 3 ppm O_3	t = 6	19 b	6.4 c	39 b	15 b	12 cd	3.0 bc	4.4 c	nd
	t = 12	18 b	6.1 c	39 b	14 b	11 de	3.0 bc	4.4 c	nd
	t = 24	13 b	4.9 c	23 b	10 b	6.1 e	1.6. d	1.0 c	nd
	P	< 0.0001	< 0.0001	< 0.0001	< 0.0001	< 0.0001	< 0.0001	< 0.0001	ns

Values are means of three replicates (*n* = 3); nd = not detected (i.e. < 1 µg/L). Different letters (within columns) indicate statistical significance (*p* = 0.05, one way ANOVA); ns = not significant. Post-harvest smoke treatments were applied on consecutive days, but smoke density was lower on the second day of treatment due to increased wind.

Table S7. Concentration (mg/g) of anthocyanins in control and smoke-affected grapes, with and without post-harvest ozone treatment.

Treatment		Cya-3-*O*-glc	Pet-3-*O*-glc	Peo-3-*O*-glc	Mal-3-*O*-glc	Del-acet-glc	Pet-acet-glc	Peo-acet-glc	Mal-acet-glc	Del-coum-glc	Cya-coum-glc & Mal-caff-glc	Pet-coum-glc	Peo-coum-glc	Mal-3-coum glc
smoke no O_3	t = 6	0.07 ± 0.00	0.12 ± 0.00	0.15 ± 0.01	0.42 ± 0.01	0.02 ± 0.00	0.02 ± 0.00	0.03 ± 0.00	0.10 ± 0.00	0.02 ± 0.00	0.01 ± 0.00	0.02 ± 0.00	0.03 ± 0.00	0.08 ± 0.00
	t = 12	0.05 ± 0.01	0.10 ± 0.01	0.13 ± 0.01	0.40 ± 0.01	0.02 ± 0.00	0.02 ± 0.00	0.03 ± 0.00	0.10 ± 0.00	0.01 ± 0.00	0.01 ± 0.00	0.02 ± 0.00	0.03 ± 0.00	0.08 ± 0.00
	t = 24	0.06 ± 0.00	0.11 ± 0.01	0.13 ± 0.01	0.43 ± 0.02	0.02 ± 0.00	0.02 ± 0.00	0.03 ± 0.00	0.11 ± 0.01	0.02 ± 0.00	0.01 ± 0.00	0.02 ± 0.00	0.03 ± 0.00	0.08 ± 0.00
smoke 1 ppm O_3	t = 6	0.07 ± 0.01	0.13 ± 0.01	0.17 ± 0.01	0.44 ± 0.03	0.03 ± 0.00	0.03 ± 0.00	0.03 ± 0.00	0.11 ± 0.00	0.02 ± 0.00	0.01 ± 0.00	0.02 ± 0.00	0.04 ± 0.01	0.07 ± 0.00
	t = 12	0.07 ± 0.01	0.11 ± 0.01	0.16 ± 0.01	0.40 ± 0.02	0.02 ± 0.00	0.03 ± 0.00	0.03 ± 0.00	0.10 ± 0.00	0.02 ± 0.00	0.01 ± 0.00	0.02 ± 0.00	0.04 ± 0.01	0.07 ± 0.01
	t = 24	0.09 ± 0.01	0.14 ± 0.01	0.20 ± 0.03	0.45 ± 0.04	0.03 ± 0.00	0.03 ± 0.00	0.04 ± 0.00	0.11 ± 0.01	0.02 ± 0.00	0.01 ± 0.00	0.02 ± 0.00	0.04 ± 0.01	0.08 ± 0.01
	p	0.0006	0.006	0.002	ns	0.004	ns	0.002	ns	ns	ns	0.003	ns	ns
smoke no O_3	t = 6	0.06 ± 0.01	0.09 ± 0.00	0.13 ± 0.01	0.32 ± 0.01	0.02 ± 0.00	0.02 ± 0.00	0.03 ± 0.00	0.09 ± 0.00	0.01 ± 0.00	0.01 ± 0.00	0.02 ± 0.00	0.03 ± 0.00	0.07 ± 0.00
	t = 12	0.06 ± 0.02	0.09 ± 0.00	0.13 ± 0.01	0.33 ± 0.03	0.02 ± 0.00	0.02 ± 0.00	0.03 ± 0.00	0.09 ± 0.01	0.02 ± 0.00	0.01 ± 0.00	0.02 ± 0.00	0.03 ± 0.00	0.07 ± 0.01
	t = 24	0.06 ± 0.01	0.08 ± 0.01	0.12 ± 0.01	0.31 ± 0.01	0.02 ± 0.00	0.02 ± 0.00	0.03 ± 0.00	0.08 ± 0.00	0.01 ± 0.00	0.01 ± 0.00	0.02 ± 0.00	0.03 ± 0.00	0.06 ± 0.00
smoke 3 ppm O_3	t = 6	0.04 ± 0.00	0.08 ± 0.01	0.11 ± 0.01	0.31 ± 0.01	0.02 ± 0.00	0.02 ± 0.00	0.03 ± 0.00	0.10 ± 0.00	0.01 ± 0.00	0.01 ± 0.00	0.01 ± 0.00	0.03 ± 0.00	0.07 ± 0.00
	t = 12	0.05 ± 0.00	0.08 ± 0.00	0.11 ± 0.00	0.30 ± 0.02	0.02 ± 0.00	0.02 ± 0.00	0.03 ± 0.00	0.09 ± 0.01	0.01 ± 0.00	0.01 ± 0.00	0.01 ± 0.00	0.03 ± 0.00	0.06 ± 0.01
	t = 24	0.05 ± 0.00	0.08 ± 0.00	0.11 ± 0.00	0.31 ± 0.00	0.02 ± 0.00	0.02 ± 0.00	0.03 ± 0.00	0.09 ± 0.00	0.01 ± 0.00	0.01 ± 0.00	0.02 ± 0.00	0.03 ± 0.00	0.07 ± 0.00
	p	ns	0.04	0.03	ns	ns	ns	ns	ns	0.006	ns	ns	ns	ns

Values are means of three replicates (n = 3) ± standard deviation measured as malvidin 3-glucoside equivalents. Different letters (within columns) indicate statistical significance (p = 0.05, one way ANOVA); ns = not significant. Del = delphinidin; Cya= cyanidin; Pet= petunidin; Peo = peonidin; Mal – malvidin; glc = glucoside; acet = acetyl; coum = coumaroyl; caff = caffeoyl. Post-harvest smoke treatments were applied on consecutive days, but smoke density was lower on the second day of treatment due to increased wind.

Table S8. Aroma and palate attributes used in sensory analysis of wines.

Attributes	Definition
Aroma	
fruit	intensity of the overall fruit aroma
smoke	perception of any type of smoke aroma, including smoked meat/bacon, toasty, charry, cigar-box, estery
cold ash	burnt aroma associate with ashes, including ashtray, tarry, campfire
earthy	any aroma associated with musty, dusty, wet-wood, barnyard, mushroom-like, dank, moldy, stagnant, stale
medicinal	aromatic characteristic of Band-Aids, disinfectant-like, including cleaning products, solvents, chemicals
burnt rubber	perception of burnt rubber-like aromas
Palate	
fruit	intensity of the overall fruit flavor
smoky	perception of smoke flavor, including bacon and smoked meat
ashy aftertaste	length of taste associated with residue of ashtray perceived in the mouth after expectorating, including coal ash, ashtray, tarry, acrid, campfire
woody aftertaste	length of taste associated with woody residue, includes wood, oak, pencil shavings
metallic	the ‘tinny’ flavor associated with metals
acidity	intensity of sour/acid taste
hotness	intensity of warmth/heat due to ethanol
bitterness	intensity of bitter taste, bitter aftertaste
drying	intensity of drying, puckering mouthfeel
